# The Role of Phytochemicals in the Inflammatory Phase of Wound Healing

**DOI:** 10.3390/ijms18051068

**Published:** 2017-05-16

**Authors:** Ahmed Shah, Saeid Amini-Nik

**Affiliations:** 1 Faculty of Medicine, University of Toronto, Toronto, ON M5S 1A8, Canada; ahm.shah@mail.utoronto.ca; 2 Department of Surgery, University of Toronto, Toronto, ON M5S 1A8, Canada; 3 Department of Laboratory Medicine and Pathobiology (LMP), University of Toronto, Toronto, ON M5S 1A8, Canada; 4 Sunnybrook Research Institute, Sunnybrook Health Science Centre, Toronto, ON M5S 1A8, Canada

**Keywords:** phytochemicals, inflammatory cytokines, wound healing, burns, chronic wounds, wound infections, hypertrophic scarring, curcumin, honey, *Terminalia*

## Abstract

Historically, plant-based products have been the basis of medicine since before the advent of modern Western medicine. Wound dressings made of honey, curcumin and other phytochemical-rich compounds have been traditionally used. Recently, the mechanisms behind many of these traditional therapies have come to light. In this review, we show that in the context of wound healing, there is a global theme of anti-inflammatory and antioxidant phytochemicals in traditional medicine. Although promising, we discuss the limitations of using some of these phytochemicals in order to warrant more research, ideally in randomized clinical trial settings.

## 1. Introduction

Wounds are disruptions to the continuity of cells due to a physical, chemical, thermal, infectious or immunological injury to the skin. Effective wound healing is defined by the restoration of functional tissue integrity. Proper wound healing is achieved by adequate activation and infiltration of inflammatory cells, neutrophils and macrophages, which produce pro-inflammatory cytokines such as tumor necrosis factor α (TNF-α) and interleukin-I (IL-I) [1]. These inflammatory cytokines result in the activation of growth factors such as transforming growth factor (TGF)-β, and several fibroblast growth factors, resulting in the proliferation and infiltration of activated fibroblasts to the wound site [1]. However, these natural healing processes can be impaired with aging, obesity, and endocrine abnormalities such as diabetes mellitus [2]. While the proper level of each cytokine is essential for healing, aberrant levels of inflammatory cytokines result in excessive fibroblast proliferation causing hypertrophic scarring, which can cause significant disfigurement of the skin.

Recently, there has been a growing interest in phytochemicals, which are plant-based products and traditional therapies, in both developing and developed countries. Although it is imperative to promote the value of all therapies, whether they are synthetic or natural, the increasing demand of phytochemical-based therapies warrants investigation of these products. Recently, there has been growing evidence of the potential applications of topical phytochemicals for the enhancement of both acute and chronic wound healing. Considering the growing evidence in favour of some phytochemical products, we discuss how phytochemicals belonging to natural products such as *Curcuma longa*, honey, and *Terminalia* species affect the inflammatory microenvironment to enhance wound healing.

## 2. The Role of Inflammation in Wound Healing

### 2.1. Acute Wounds

Acute wounds primarily refer to penetrative injuries to the skin and the underlying organs due to laceration, stab or excoriation. Acute wounds can also include burns, which are a broad category of cutaneous injuries that can result from heat, cold, chemical, or radiation exposure. However, the healing outcome of burn injuries is different from penetrative injuries, as the former results in significantly more fibrosis and scarring than the latter [3].

The process of wound healing is divided into four distinct, yet overlapping stages: hemostasis, inflammation, proliferation and remodeling [4]. Immediately following a cutaneous injury, hemostasis is achieved with the activation of platelets resulting in clot formation [1], which essentially acts as a temporary wound closure mechanism. Within hours of the injury, neutrophils begin homing to the site of injury due to the effects of platelet-derived growth factors (PDGF), TGF-β and fibroblast growth factor (FGF), which act as potent chemotactic agents for neutrophils [1]. Neutrophils act as the first-line defense against microbial invasion and foreign debris, which is cleared by phagocytosis. Natural killer (NK) cells also infiltrate the wound in early inflammation along with neutrophils and regulate the production of important monocyte cytokines [5]. Beyond 72 h post-injury, activated macrophages replace neutrophils as the dominant cell type in the wound site [6]. Macrophages release pro-inflammatory cytokines including TNF-α and IL-1 [7], which in turn activate the nuclear-factor κB (NF-κB)-mediated release of pro-inflammatory cytokines and the release of matrix metalloproteinases (MMPs) [8]. MMPs degrade damaged cells and also release growth factors from the extracellular matrix.

Apart from creating a pro-inflammatory microenvironment, macrophages also release growth factors including vascular endothelial growth factor (VEGF), TGF-β, basic FGF (bFGF), PDGF, and keratinocyte growth factor (KGF), which promote keratinocyte and fibroblast migration, proliferation, and angiogenesis [7]. This marks the proliferative stage of wound repair.

T lymphocytes migrate to the wound site on the fifth day post-injury, which correlates with the late inflammation and early proliferation phase, reaching their peak on day 7 [9]. Although T lymphocytes migrate after neutrophils and macrophages, their contribution to wound repair has been previously noted. For instance, global CD4+ T lymphocyte depletion in animal models is associated with decreased wound-breaking strength and collagen content [9]. Histologically, CD4+ T-helper 1 (Th1) cells are the most abundant lymphocytes in human wound sites [10]. Th1 cells secrete cytokines that activate macrophages, which may be another major contributory role of T lymphocytes in wound healing [11]. Furthermore, CD40 ligand-expressing T cells directly interact with CD40-expressing macrophages, fibroblasts and keratinocytes, thereby modulating their wound healing response [12].

Although B lymphocytes have been found in wound tissue [10], their role in wound healing is still unclear. It has been suggested that B lymphocytes could be involved in the recruitment of neutrophils and macrophages to the wound site [13]. This is demonstrated by the fact that the loss of CD19 expression in mice results in decreased infiltration of neutrophils and macrophages to the wound, and decreased re-epithelialization and granulation tissue formation [13].

Fibroblasts are crucial for producing type III collagen fibers [7]. Proliferation, migration, and differentiation of keratinocytes are crucial for wound re-epithelialization and the restoration of the epidermal barrier [14]. The remodeling phase, which can span several months, is a period of reduced inflammation and reorganization of collagen fibers from immature type III to mature type I, thus closely resembling normal tissue [6].

### 2.2. Excessive Healing

While in an optimal condition, human skin can heal with a minimal scar. However, in certain conditions the inflammatory/proliferation phase of wound healing lasts for a longer period, leading to excessive healing such as that observed in hypertrophic scarring. The exact pathophysiology of hypertrophic scarring remains elusive, but it appears to be due to a prolonged inflammatory period resulting in increased fibroblast activity and excessive deposition of extracellular matrix components such as collagen [15]. Hypertrophic scarring is a common occurrence in post-surgical wounds and burn injuries, the latter having an incidence of 70% [3,16].

There are several differences between normal wound healing and hypertrophic scarring (Table 1). Burn wounds have significantly more TGF-β1 being produced [17]. This acts as a chemotactic to activated fibroblasts and also causes fibroblast differentiation to myofibroblasts. In hypertrophic scarring, there is also an abundance of differentiated myofibroblasts, which are potent wound contractors but also lead to hypertrophic scarring [17]. Furthermore, increased collagen synthesis and decreased collagenase activity is also noted in hypertrophic scars as compared to normal wound healing [3]. This means that, in hypertrophic scars, there is a higher abundance of immature type III collagen fibers and less prevalence of mature type I collagen fibers [18]. Immunosuppressive drugs including triamcinolone acetonide, an intra-lesionally administered corticosteroid, and 5-fluorouracil, have been successfully used for the treatment of both hypertrophic scar formation and keloids [19]. These immunomodulatory agents have been shown to suppress fibroblast proliferation and collagen production [19]. This suggests further that the inflammatory response is associated with this pathologic excessive healing.

Apart from the prolonged inflammatory response, recent evidence suggests that the type of immune response is crucial for the formation of hypertrophic scarring or keloid formation. It has been shown that T-helper (Th) cells are major immune mediators in the inflammatory phase. However, if Th cells differentiate into Th2 cell types, they result in fibrogenesis and hypertrophic scarring, whereas Th1 cell types, which produce interferon-γ (IFN-γ), decrease tissue fibrosis [20]. Thus, interferon therapies have shown promise in recent years [21]. However, interferon therapy has not replaced intra-lesional corticosteroids as first-line therapy, as they are very painful and very costly [21].

### 2.3. Deficient Healing: Non-Healing Wounds

In contrast to acute wounds, where inflammation plays a crucial role in wound repair, chronic non-healing wounds could result from the aberrant inflammatory response (Table 1). For instance, diabetic foot ulcers are characterized by a hyper-inflammatory response characterized by dysregulated and sustained infiltration of neutrophils and macrophages [22]. There is evidence that an abundance of neutrophils in wounds can negatively affect wound repair because of their destructive nature of its function [23]. Due to the abundance of neutrophils, chronic wounds become very proteolytic environments composed primarily of host-derived proteases [24]. Significantly elevated inflammatory cytokines and collagenases such as MMP8 [25] and gelatinases including MMP2 and MMP9 [26] lead to excessive tissue destruction. This pro-inflammatory and destructive microenvironment and is also exemplified by the reduced levels of growth factors found in these wounds [27]. Furthermore, both neutrophils and macrophages produce radical oxygen species as a defense mechanisms against invading microbes [28], which can aggravate the inflammation and cytotoxicity of the microenvironment and thus delay wound healing [29].

Macrophages found in chronic diabetic wounds demonstrate sustained pro-inflammatory phenotypes [30]. Studies have shown that chronic diabetic wounds have dysregulated TNF-α signaling and NF-κB overexpression. Thus, therapies that can target the mechanisms behind hyper-inflammation in chronic wounds would be ideal for enhancing wound repair in conditions such as diabetic ulcers.

## 3. Phytochemicals and Traditional Therapies in Wound Healing

Plant-based products have been used to treat wounds for centuries worldwide. Recently, fascinating new evidence has emerged to highlight the mechanisms of some of these traditional therapies. The most common mechanisms behind phytochemical-mediated enhanced wound healing are their antioxidant, anti-inflammatory, and anti-microbial effects.

The process of inflammation mediated by neutrophils and macrophages leading to the destruction of foreign debris and microorganisms results in the production of radical oxygen species. Overproduction of radical oxygen species due to dysregulated immune response seen in chronic non-healing wounds may exacerbate the condition by further promoting tissue damage and delaying wound healing [29]. Therefore, compounds that act as free-radical neutralizers or possess antimicrobial properties may play an important role in enhancing wound healing. Several traditional plant-based therapies have been shown to possess antioxidant activity and also promote wound healing in in vitro studies [31].

### 3.1. Curcumin

One of the most extensively studied phytochemicals for wound healing is curcumin, which is a chemical compound present in the Asian spice turmeric or *Curcuma longa*. Apart from usage in Indian and Chinese cuisine, turmeric has been used topically for cutaneous wounds including ulcers, traditionally in the Indian subcontinent [32]. The main mechanism by which curcumin impacts wound healing is through its anti-inflammatory properties. In vitro studies have demonstrated the suppression of TNF-α and IL-1 production by human macrophages [33]. Moreover, curcumin is also a potent inhibitor of phosphorylase kinase (PhK) and NF-κB activation [34,35]. This makes curcumin a great phytochemical candidate for the treatment of hyper-inflammatory wounds such as chronic diabetic wounds and burns.

Since curcumin is a hydrophobic compound, its dermal delivery is minimal [36]. Consequently, different formulations have been created to enhance topical usage of curcumin such as gels [37], polymetric bandages [38], collagen films [39], alginate foams [40]. Moreover, lipid-core nanocapsule (LCN) significantly enhances the dermal delivery of curcumin [41]. Thus, LCN-based delivery systems of curcumin show significant promise for topical applications of curcumin. More recently, the use of curcumin-loaded chitosan nanoparticles [42], hyalusomes [36] and hydrogels [43] also show promise in the cutaneous usage of curcumin in the future.

#### 3.1.1. Curcumin and Chronic Wounds

Chronic wounds are hyper-inflammatory and highly proteolytic environments. Thus, controlling this dysregulated inflammation is crucial to ensure adequate wound healing. Topical curcumin shows promise in the management of chronic non-healing wounds. Topical curcumin treatment of wounds of streptozotocin-induced diabetic rats showed faster re-epithelialization, increased migration of fibroblasts to the wound bed, improved vascularization and significantly higher collagen content than control animals [44]. Diabetic wounds have diminished angiogenic potential, thus prolonging wound healing [45]. It is interesting to note that topical curcumin treatment on the wounds of diabetic rats also showed enhanced angiogenesis demonstrated by significant upregulation in VEGF [46].

**Figure 1:**
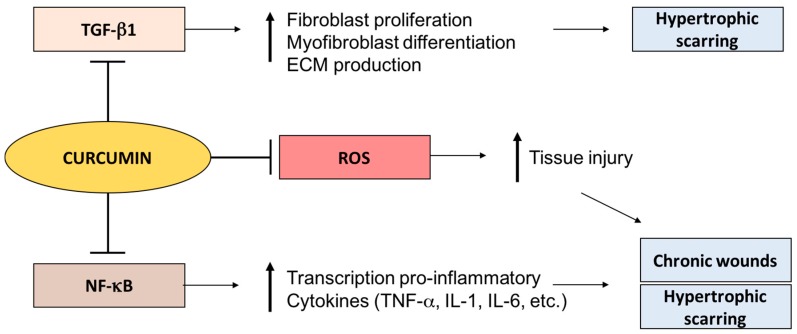
The anti-inflammatory effects of curcumin in the context of wound healing. Curcumin inhibits transforming growth factor (TGF)-β expression, thereby it may decrease TGF-β1-mediated fibroblast proliferation and excessive extracellular matrix (ECM) production seen in hypertrophic scars. Moreover, curcumin also inhibits nuclear-factor κB (NF-κB)-mediated transcription of pro-inflammatory cytokines, thereby reducing inflammation seen in chronic wounds and hypertrophic scars. It also sequesters radical oxygen species (ROS) through its antioxidant activity. T bars are used as a symbol of inhibition, the horizontal arrows are used as a symbol of facilitation, and bolded vertical arrows signify an increase (upward) or decrease (downward) end result.

Chronic wounds also have high levels of radical oxygen species such as hydrogen peroxide and superoxides, which can be extremely detrimental to wound healing. Curcumin has potent antioxidant action in cutaneous wounds. In rat excisional wound models, transdermal application of curcumin reduced the damage caused by hydrogen pyroxide on fibroblasts and keratinocytes [47]. In another study, topical application of curcumin in excised wounds in rats not only showed faster epithelialization than control but also showed reduced lipid peroxidase levels, in addition to the significant upregulation of anti-oxidant enzymes such as catalase, glutathione peroxidase and superoxide dismutase (Figure 1) [48]. Interestingly, in inflammatory and oxidative stress-induced conditions such as vitiligo [49], topical curcumin has also shown promise in clinical trials [50]. It will be interesting to see the efficacy of topical curcumin on human diabetic wounds.

#### 3.1.2. Curcumin and Hypertrophic Scarring

In hypertrophic scarring and keloids, there is an abundance of TGF-β1 expression, fibroblast proliferation, and excess collagen and extracellular matrix (ECM) synthesis [51]. Apart from being a potent inhibitor of NF-κB, curcumin inhibits TGF-β1 signaling in keloid fibroblasts and also diminishes ECM production (Figure 1) [52]. Moreover, curcumin has also been shown to inhibit TGF-β1 signaling in scleroderma, another fibrotic skin disease [53]. Curcumin also suppresses the proliferation of keloids and hypertrophic scar-derived fibroblasts in vitro [54]. Therefore, topical curcumin may show promise in hypertrophic scar prevention.

The use of curcumin gel in six case reports of post-surgical patients has shown promise, with wound healing achieved with minimal scarring [37]. However, randomized controlled trials would be necessary for determining the true efficacy of these therapies. Considering that modern first-line therapy for hypertrophic scars and keloid prevention is intra-lesional corticosteroid injections, which have potent immunosuppressive effects, perhaps curcumin may show some efficacy in human trials.

### 3.2. Honey

Honey has been a component of traditional medicine in diverse parts around the globe. One of its most common usages has been topical treatment for chronic wounds and burns [55]. Since the primary components of honey are plant-based, honey has been extensively studied for its phytochemical properties [56,57]. Studies have found that types of honey may differ in their wound-healing properties depending on their phytochemical profile, which depends on their floral sources [55].

Honey has some antibacterial effects [58]. This is particularly important for burns and chronic wounds. Although any other type of wound can be affected by infections because the loss of the skin barrier allows for microbial penetration, chronic wounds and burns are particularly vulnerable to infections. The avascular wound bed of burn injuries provides an ideal environment for microbial growth within and may spread beyond the wound site [59]. Apart from its antimicrobial effects, honey also has many immunomodulatory effects that are useful for the management of chronic wounds. Honey is also shown to promote angiogenesis and fibroblast proliferation in human clinical trials [60].

#### 3.2.1. Honey and Chronic Wounds

**Figure 2:**
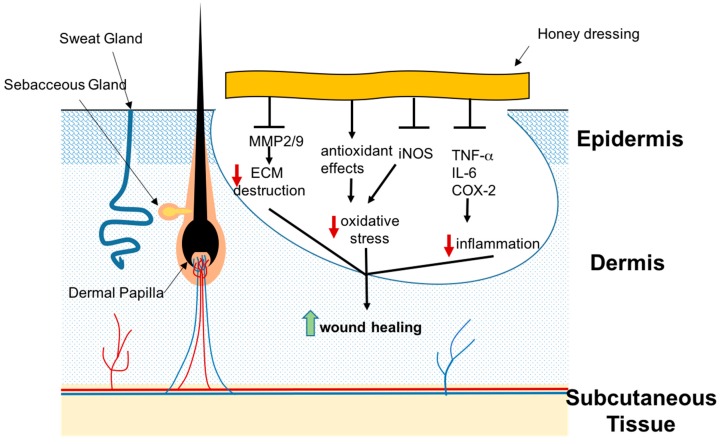
The anti-inflammatory effects of honey in chronic wounds. Honey inhibits matrix metalloproteinases (MMPs), thereby diminishing their ECM destructive effects. By inhibiting inducible nitric oxide synthase (iNOS) compounded by its antioxidant effects, it reduces oxidative stress-mediated tissue injury. Moreover, by inhibiting inflammatory proteins, it has an overall anti-inflammatory effect in chronic wounds. This is necessary to achieve wound healing in chronic, hyperinflammatory wounds such as diabetic ulcers. T bars are used as a symbol of inhibition, the bolded black arrows are used as a symbol of facilitation, and vertical coloured arrows signify an increase (upward) or decrease (downward) end result. Unbolded arrows are used for labelling.

The antibacterial effects of honey, which include both bacteriostatic and bacteriocidal activities, make it of use to eliminate pathogens whilst having a moist environment favorable to wound healing [61]. In order to achieve wound healing in diabetic ulcers, debridement of old dead cells and tissues is crucial. Honey contains protease enzymes that facilitate debridement of the wounds [61]. However, since chronic wounds have a hyper-inflammatory microenvironment, without controlling inflammation there is little chance of achieving wound repair. Honey exerts its anti-inflammatory effects by the inhibition of cyclooxygenase-2 (COX-2), inducible nitric oxide synthase (iNOS), TNF-α and IL-6 expression (Figure 2) [62]. Honey is also shown to inhibit MMP9, which may help reduce the degradation of ECM in chronic wounds (Figure 2) [63]. Caffeic acid phenol ester (CAPE), a polyphenol found in honey, has exhibited the suppression of MMP2 and MMP9 in fibrosarcoma cells [64]. A similar suppression of MMP2 and MMP9 by honey has been shown in other cell types such as human glioblastoma multiforme cell lines [65]. The inhibition of MMP2 and MMP9 is of particular interest because there is increased expression of collagenases and gelatinases in chronic wounds, which result in impaired healing. Furthermore, honey contains various compounds including flavonoids, phenolic acids, catalase, peroxidase, carotenoids, and ascorbic acid, which possess antioxidant properties that can counteract the abundance of free-radicals found in chronic wounds (Figure 2) [66,67].

The efficacy of honey on chronic wounds is well-documented in clinical trials. A study consisting of 59 patients, most with wounds and ulcers that failed to respond to conventional treatment, showed rapid epithelialization after treatment with topical honey [68]. In another study consisting of 30 infected diabetic foot wounds that were treated with honey dressings, complete healing was achieved in 43.3% of the patients, while only 6.7% of ulcers did not respond to treatment [69]. An analysis of 17 randomized controlled trials and five clinical trials showed positive outcomes for the use of honey as a wound dressing [70].

#### 3.2.2. Honey and Burns

Honey has been used for burns in various ancient societies. Greek and Roman physicians, for instance, used honey for the treatment of burn wounds [71]. Recently, the usage of honey in the context of burns is coming to light again.

In rat models of partial-thickness burn injuries, honey formulations shortened the period of epithelization and increased wound contraction compared to vehicle controls [72]. In humans, a systematic review of randomized controlled trials of eight studies comparing the efficacy of honey to silver sulphadiazine-impregnated gauze showed that honey had a superior healing effect [73]. However, this was limited to superficial and partial thickness burns only.

Furthermore, honey has been shown to be protective against hypertrophic scarring in the context of burn wounds. In a randomized-controlled trial examining the effects of honey in comparison to silver sulfadiazine in 104 patients with superficial burns, there was a significantly lower incidence of hypertrophic scarring and post-burn contracture in the honey-treated group in comparison to the silver sulfadiazine-treated group [74].

### 3.3. Terminalia Genus

The genus *Terminalia* is comprised of over 200 species of trees, and a variety of these species have been historically used as traditional medicine in Asia, Africa, and Australia. Some *Terminalia* species have been reported to have wound-healing properties, antioxidant and antimicrobial activity [75]. Studies examining *Terminalia* species used in Ayurvedic medicine, such as *T. arjuna* and *T. chebulu*, have been shown to have wound-healing effects in rat excision wound models [76,77].

**Figure 3:**
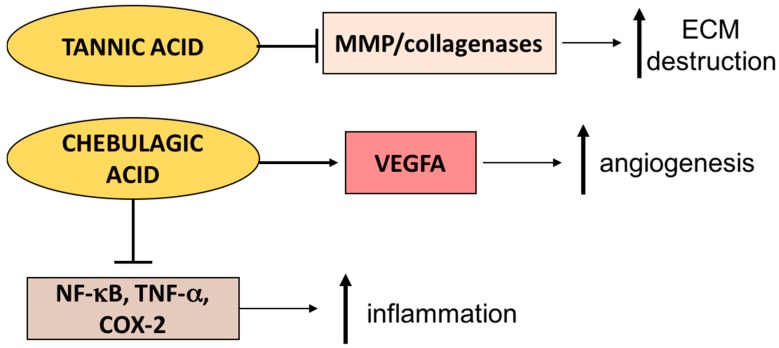
The anti-inflammatory effects of phytochemicals derived from *Terminalia* species in wounds. Tannic acid extracted from *T. arjuna* inhibits MMPs, thus reducing MMP-mediated ECM destruction. Chebulagic acid (CA) derived from *T. chebula* inhibits pro-inflammatory pathways mediated by NF-κB, tumor necrosis factor α (TNF-α), and cyclooxygenase-2 (COX-2). Moreover, CA also increases vascular endothelial growth factor (VEGF) A-mediated angiogenesis. T bars are used as a symbol of inhibition, the horizontal arrows are used as a symbol of facilitation, and bolded vertical arrows signify an increase (upward) or decrease (downward) end result.

*T. arjuna* is composed of tannins, which have been shown to possess antioxidant and anti-inflammatory properties. Moreover, tannic acid (TA), a plant polyphenol, has been shown to stabilize collagen and elastin in the extracellular matrix. This is achieved by inhibiting MMP collagenases while enhancing collagen cross-linking (Figure 3) [78,79]. Moreover, collagen scaffolds prepared by TA cross-linking have more tensile strength and greater resistance to collagenase-mediated degradation than collagen scaffolds without TA [80]. Since chronic wounds are characterized by hyper-inflammation and high collagenase activity, the effects of *T. arjuna* topical treatment on human diabetic wounds is worth investigating.

*T. chebula* is reported to enhance extracellular matrix deposition in granulation tissues in rat excision wound models [81]. *T. chebula* extracts have been shown to enhance keratinocytes and fibroblasts growth in vitro [82]. Furthermore, rat wounds treated with *T. chebula* had significantly reduced lipid peroxide levels, suggesting the antioxidant role of *T. chebula* topical treatment [81], which was confirmed by electronic spin resonance (ESR)-2,2,-diphenyl-1-picrylhydrazyl (DPPH) assays [81,82]. Tannins extracted from *T. chebula* also promote angiogenesis in wounds of rat models shown by the upregulation of vascular endothelial growth factor (VEGF) A expression and increased new vessel formation in the inflammatory phase (Figure 3) [76]. It is also possible that the wound-healing effects of *T. chebula* are also due to its anti-inflammatory effects. Chebulagic acid (CA), an anti-oxidant compound extracted from *T. chebula*, when cultured with macrophages in vitro, significantly suppressed NF-κB activation as well as TNF-α and COX-2 expression (Figure 3) [83]. It is possible that topical application of *T. chebula* would be beneficial in hyper-inflammatory wounds such as chronic diabetic wounds or burns. Supporting this concept, increased wound healing in streptozotocin-induced diabetic rats with the topical application of *T. chebula* extract has been shown [84]. Moreover, *T. chebula* extract accelerates wound healing in burn wounds in comparison to 1% silver sulfadiazine in rat models [85].

Historically, in South Africa and much of sub-Saharan Africa, the barks of *Terminalia sericea* have been crushed and applied topically to cutaneous wounds, skin infections and burns [78,86]. Although *Terminalia sericea* has been shown to not promote fibroblast growth, its antioxidant activity and antimicrobial activity against *Staphylococcus aureus* and *Streptococcus pyogenes* [86,87,88] have been documented. This is particularly useful in traditional methods, since the most common causes of skin infection are *Staphylococcus* and *Streptococcus* species [89]. Moreover, the mild antimicrobial activity of *Terminalia sericea* against *Pseudomonas aeroginosa* has also been reported [88].

### 3.4. Other Plant-Based Sources of Wound Care

Globally, there seems to be a theme among phytochemicals that are used for wound healing in traditional medicine. Most of them possess either anti-inflammatory or antioxidant activity. *Chromolaena odorata* is widely found in southern Asia and western Africa, and has been traditionally used for the treatment of wounds in Vietnam for many years. It has been demonstrated that *Chromolaena odorata* promotes wound contraction in in vitro models [90], and also promotes fibroblast proliferation [91]. Furthermore, *Chromolaena odorata* has been shown to have protective effects on human fibroblasts and keratinocytes against the oxidative damage of hydrogen peroxide [92]. Similarly, *Bridelia ferruginea* leaf, which has been used widely in Nigeria as a topical wound treatment, also possesses antioxidant activity and stimulates fibroblast growth [93]. *Ficus asperifolia* and *Gossypium arboreum*, wound healing plants used traditionally in Ghana, also possess the same ability [94].

Furthermore, several others phytochemicals derived from herbal sources have shown some efficacy in animal models on the treatment of burn wounds [95]. However, only a few of the vast majority of these herb-derived phytochemicals have been studied in human trials [95]. One of these exceptions is the herb *Aloe vera*, which has been studied in both animal and human-based studies. A topical gel derived from *A. vera* has shown the reduction of inflammatory cytokines TNF-α and IL-6 in rat burn models [96]. Furthermore, it also showed increased re-epithelialization in burn models of rat [97,98] and guinea pig [99]. *A. vera* has been shown to be superior to 1% silver sulphadiazine cream by promoting faster wound contraction and re-epithelization time in two randomized controlled trials of 50 [100] and 27 [101] partial-thickness or second-degree burn patients. These promising results call for more studies with a greater number of patients to investigate not only the efficacy in wound healing of *A. vera* and other herb-based phytochemicals, but also the propensity for hypertrophic scarring compared to conventional therapies.

### 3.5. Therapies Combining Phytochemicals with Modern Wound Dressings

Biopolymer-based hydrogel dressings are known for their hydrophilicity, higher swelling capacity and increased biocompatibility compared to conventional wound dressings [102]. Hydrogel dressings have been shown to be superior to conventional dressings as they can maintain a moist environment which enhances wound healing [103]. Moreover, hydrogels can be employed as drug delivery systems along with anti-bacterial applications [103]. In accordance with the fact that silver-based compounds have been used in the treatment of burns and infections, the efficacy of hydrogels with silver-based nanoparticles has also been studied and established [104,105]. Interestingly, there is growing evidence of the efficacy of phytochemicals in hydrogel-based dressings.

The combination of silver-curcumin hydrogel films has been shown to be superior to films employing silver and curcumin alone in inhibiting the growth of *Escherichia coli* (*E. coli*) [106]. Furthermore, curcumin-loaded poly(epsilon-caprolactone) dressings demonstrate enhanced wound closure in streptozotocin-treated diabetic mice [107]. Thus, the application of curcumin in modern dressings has enormous applications in the context of chronic wounds and burn.

Similarly, honey has also been employed in hydrogels. In rat burn models, honey-based hydrogels have been shown significantly enhance wound closure and accelerate re-epithelialization compared to control hydrogel dressings [108]. Furthermore, honey-based carboxymethyl cellulose (CMC) hydrogels have also been shown to have enhanced antimicrobial effects against *Staphylococcus aureus* and *E. coli* as well as accelerated wound closure in diabetic rat models [109].

### 3.6. Effects of Phytochemicals on Stem Cells

Recently there has been growing evidence of phytochemicals and their effects on stem cells, especially cancer stem cells [110]. Studies are demonstrating that the ability of phytochemicals to inhibit tumor formation is in part by targeting stem cell signaling pathways including the Sonic hedgehog (Shh), Wingless (Wnt)/β-catenin and Notch signaling pathways [110,111,112]. Although Shh and Notch signaling have some roles in wound repair, they are relatively unexplored if compared to the role of the Wnt/β-catenin signaling pathway [113]. Interestingly, the Wnt/β-catenin signaling pathway is activated during the proliferative phase of wound repair and is responsible for regulating dermal fibroblast activity and proliferation during wound healing [114,115].

**Figure 4:**
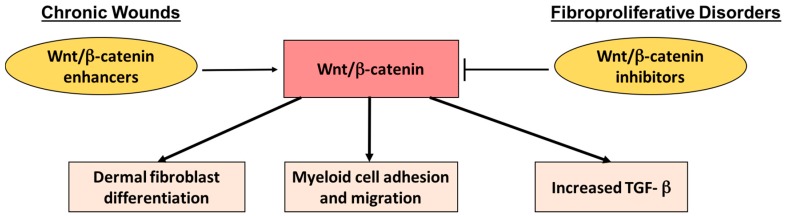
Proposed mechanism for the modulation of Wingless (Wnt)/β-catenin signaling in the context of fibroproliferative disorders and chronic wounds. Increased Wnt//β-catenin signaling is associated with increased dermal fibroblast differentiation from local mesenchymal stem cells and Pax7-positive muscle stem cells, myeloid cell adhesion, and migration and increased TGF-β1 production. For fibroproliferative disorders, Wnt/β-catenin inhibitors can be utilized to inhibit excessive collagen synthesis and deposition. On the other hand, for chronic wounds, Wnt/β-catenin enhancers could be utilized to enhance wound healing. T bars are used as a symbol of inhibition, and the horizontal arrows are used as a symbol of facilitation.

The role of stem cells and their signaling pathways in the context of wound healing is well-established. Our group has shown that Wnt/β-catenin signaling in the proliferative phase of wound healing stimulates local mesenchymal stem cells and Pax7 muscle stem cells to differentiate to dermal fibroblasts and contribute to wound healing [111,113,114,116,117]. Moreover, our group has shown that β-catenin signaling also regulates myeloid cell adhesion to fibroblasts and migration to wound sites [116]. Macrophages lacking β-catenin produce less TGF-β1 [116], low levels of which is correlated with impaired myofibroblast differentiation [118]. However, dysregulated β-catenin signaling results in TGF-β-mediated fibroproliferative disorders such as hypertrophic scarring [119,120]. Therefore, pharmacological therapies and phytochemicals that modulate β-catenin activity could be used for enhancing wound healing. β-Catenin inhibitors may be useful for managing fibroproliferative disorders such as keloids and hypertrophic scarring, whereas β-catenin enhancers may be used for conditions with impaired wound healing such as chronic diabetic wounds (Figure 4). Several studies have identified phytochemicals that inhibit Wnt/β-catenin signaling, including curcumin, tea polyphenols, and sulforaphanes found in cruciferous vegetables [111]. Therefore, future studies should examine the use of β-catenin-modulating phytochemicals in wound healing.

## 4. Discussion and Conclusions

**Figure 5:**
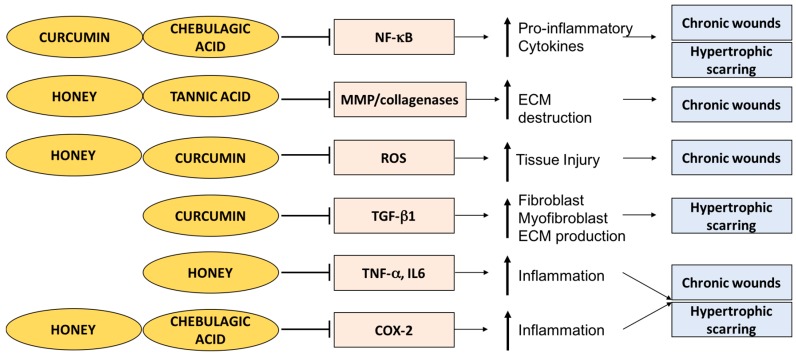
Summary of anti-inflammatory effects of curcumin, honey, and phytochemicals derived from *Terminalia* species in the context of wound healing. T bars are used as a symbol of inhibition, the horizontal arrows are used as a symbol of facilitation, and bolded vertical arrows signify an increase (upward) or decrease (downward) end result.

In this review, we show that in the context of wound healing there is a global theme of anti-inflammatory and antioxidant phytochemicals in traditional medicine. Whether it be turmeric, honey, *Terminalia sericea*, *Chromolaena odorata* or *Bridelia ferruginea*, they all possess anti-inflammatory and antioxidant potential (Figure 5). We show that curcumin, honey and phytochemicals derived from *Terminalia* are intricately involved in the inflammatory phase of wound healing (Table 2). Phytochemicals such as curcumin have potent anti-inflammatory effects such as the inhibition of NF-κB activation (Figure 5), making it a potential candidate for future trials for a topical treatment for chronic wounds and for the prevention of hypertrophic scarring and keloids. However, the studies investigating curcumin effects are primarily on rat wound models. Future studies show a focus on enhancing the dermal delivery of topical curcumin and developing human trials to evaluate the efficacy of curcumin on human wound repair. Moreover, honey has shown great therapeutic potential in trials as wound dressings for chronic diabetic ulcers and superficial and partial-thickness burn injuries. The development of modern wound dressings using hydrogels and utilizing honey or curcumin, or a combination, could be of interest in the future.

Compounds derived from *Terminalia* genus species have great wound-healing potential. Chebulagic acid derived from *T. chebula* has potent anti-inflammatory effects such as the inhibition of COX-2 and NF-κB (Figure 5). Also, tannic acid strengthens collagen scaffolds and inhibits the MMP-mediated destruction of ECM (Figure 5). It will be interesting to see the efficacy of these compounds on wound healing in humans.

The role of phytochemicals in stem cell signaling has yet to be explored in the context of wound healing. Considering the important role of Wnt/β-catenin signaling in both wound healing and the formation of hypertrophic scarring, it is important to explore the role of phytochemicals that modulate this pathway. Perhaps utilizing phytochemicals that suppress β-catenin signaling may be a new preventative measure for the formation of hypertrophic scars and keloids.

With the exception of some phytochemical-rich products such as honey-based dressings and topical *A. vera* gels, our review highlights the reliance on animal models and the lack of human trials on phytochemicals. There is a need for studies evaluating a greater number of patients to investigate the efficacy of plant-based, herbal and traditional therapies on wound healing and scarring.

Historically, plant-based products have been the basis of medicine before the advent of modern Western medicine. Today, it is estimated that about 80% of the word relies on phytochemicals and traditional remedies for their ailments [121]. Although modern medicine has made significant advancements in therapies, many of the new therapies are either inaccessible or unaffordable to much of the world. Moreover, there is a growing demand for phytochemical-based therapies, even in the developed world, which warrants more investigations to test the efficacies for these widely used remedies.

## Figures and Tables

**Table 1:** Summary of the differences between normal, excessive, and deficient wound healing responses.

Normal	Excessive	Deficient
Adequate inflammatory response	Prolonged inflammatory phase	Dysregulated hyperinflammatory response
Adequate collagenase expression	Decreased collagenase expression	Overexpression of collagenases and gelatinases
Increased type I collagen	Increased type III collagen	Matrix metalloproteinases (MMP)-mediated destruction of extra-cellular matrix (ECM)
Minimal scarring	Hypertrophic scarring/fibrosis	Inadequate wound closure

**Table 2:** Summary of the roles of curcumin, honey and *Terminalia* phytochemicals on wound healing. Curcumin inhibits nuclear-factor κB (NF-κB) and inflammatory cytokines. Moreover, curcumin is shown to inhibit transforming growth factor (TGF)-β1 expression and Wnt/β-catenin signaling in vitro and to upregulate VEGF in vivo, highlighting its role in the proliferative phase. Honey is shown to have an anti-inflammatory role as well as to promote fibroblast and angiogenesis in clinical trials. Phytochemicals derived from *Terminalia* are shown to modulate inflammation. They are also shown to promote fibroblast and keratinocyte growth in vitro and angiogenesis in vivo, thus playing a role in the proliferative phase as well.

Phytochemical	Phase of Wound Healing	In Vitro	In Vivo (Animal)	In Vivo (Human)
Curcumin	Inflammatory phase	[33,34,35]	Rat [47,48]	Clinical Trials [50]
	Proliferation phase	[52]	Rat [46]	-
	Remodeling phase	-	-	-
Honey	Inflammatory phase	[64]	Rat [70]	RCT [66,67,68,71,72]
	Proliferation phase	-	-	RCT [72]
	Remodeling phase	-	-	-
*Terminalia*	Inflammatory phase	[76,77,78,81]	Rat [79,82,83]	-
	Proliferation phase	[80]	Rat [74]	-
	Remodeling phase	-	-	-

## References

[1] Portou M.J., Baker D., Abraham D., Tsui J. (2015). The innate immune system, toll-like receptors and dermal wound healing: A review. Vascul. Pharmacol..

[2] Guo S., Dipietro L.A. (2010). Factors affecting wound healing. J. Dent. Res..

[3] Finnerty C.C., Jeschke M.G., Branski L.K., Barret J.P., Dziewulski P., Herndon D.N. (2016). Hypertrophic scarring: The greatest unmet challenge after burn injury. Lancet.

[4] Singer A.J., Clark R.A. (1999). Cutaneous wound healing. N. Engl. J. Med..

[5] Schneider D.F., Palmer J.L., Tulley J.M., Speicher J.T., Kovacs E.J., Gamelli R.L., Faunce D.E. (2011). A novel role for NKT cells in cutaneous wound repair. J. Surg. Res..

[6] DiPietro L.A. (1995). Wound healing: The role of the macrophage and other immune cells. Shock.

[7] Rodero M.P., Khosrotehrani K. (2010). Skin wound healing modulation by macrophages. Int. J. Clin. Exp. Pathol..

[8] Han Y.P., Tuan T.L., Wu H., Hughes M., Garner W.L. (2001). TNF-α stimulates activation of pro-MMP2 in human skin through NF-κB mediated induction of MT1-MMP. J. Cell Sci..

[9] Park J.E., Barbul A. (2004). Understanding the role of immune regulation in wound healing. Am. J. Surg..

[10] Boyce D.E., Jones W.D., Ruge F., Harding K.G., Moore K. (2000). The role of lymphocytes in human dermal wound healing. Br. J. Dermatol..

[11] Stout R.D., Suttles J. (1997). T cell signaling of macrophage function in inflammatory disease. Front. Biosci..

[12] Eming S.A., Hammerschmidt M., Krieg T., Roers A. (2009). Interrelation of immunity and tissue repair or regeneration. Semin. Cell Dev. Biol..

[13] Iwata Y., Yoshizaki A., Komura K., Shimizu K., Ogawa F., Hara T., Muroi E., Bae S., Takenaka M., Yukami T. (2009). CD19, a response regulator of B lymphocytes, regulates wound healing through hyaluronan-induced TLR4 signaling. Am. J. Pathol..

[14] Pastar I., Stojadinovic O., Yin N.C., Ramirez H., Nusbaum A.G., Sawaya A., Patel S.B., Khalid L., Isseroff R.R., Tomic-Canic M. (2014). Epithelialization in wound healing: A comprehensive review. Adv. Wound Care.

[15] Wang J., Hori K., Ding J., Huang Y., Kwan P., Ladak A., Tredget E.E. (2011). Toll-like receptors expressed by dermal fibroblasts contribute to hypertrophic scarring. J. Cell. Physiol..

[16] Butzelaar L., Ulrich M.M., Mink van der Molen A.B., Niessen F.B., Beelen R.H. (2016). Currently known risk factors for hypertrophic skin scarring: A review. J. Plast. Reconstr. Aesthet. Surg..

[17] Wang J., Jiao H., Stewart T.L., Shankowsky H.A., Scott P.G., Tredget E.E. (2007). Increased TGF-β-producing CD4+ T lymphocytes in postburn patients and their potential interaction with dermal fibroblasts in hypertrophic scarring. Wound Repair Regen..

[18] Oliveira G.V., Hawkins H.K., Chinkes D., Burke A., Tavares A.L., Ramos-e-Silva M., Albrecht T.B., Kitten G.T., Herndon D.N. (2009). Hypertrophic versus non hypertrophic scars compared by immunohistochemistry and laser confocal microscopy: Type I and III collagens. Int. Wound J..

[19] Leventhal D., Furr M., Reiter D. (2006). Treatment of keloids and hypertrophic scars: A meta-analysis and review of the literature. Arch. Facial Plast. Surg..

[20] Gauglitz G.G., Korting H.C., Pavicic T., Ruzicka T., Jeschke M.G. (2011). Hypertrophic scarring and keloids: Pathomechanisms and current and emerging treatment strategies. Mol. Med..

[21] Rabello F.B., Souza C.D., Farina Junior J.A. (2014). Update on hypertrophic scar treatment. Clinics.

[22] Wetzler C., Kampfer H., Stallmeyer B., Pfeilschifter J., Frank S. (2000). Large and sustained induction of chemokines during impaired wound healing in the genetically diabetic mouse: Prolonged persistence of neutrophils and macrophages during the late phase of repair. J. Investig. Dermatol..

[23] Dovi J.V., He L.K., DiPietro L.A. (2003). Accelerated wound closure in neutrophil-depleted mice. J. Leukoc. Biol..

[24] Menke N.B., Ward K.R., Witten T.M., Bonchev D.G., Diegelmann R.F. (2007). Impaired wound healing. Clin. Dermatol..

[25] Nwomeh B.C., Liang H.X., Cohen I.K., Yager D.R. (1999). MMP-8 is the predominant collagenase in healing wounds and nonhealing ulcers. J. Surg. Res..

[26] Wysocki A.B., Staiano-Coico L., Grinnell F. (1993). Wound fluid from chronic leg ulcers contains elevated levels of metalloproteinases MMP-2 and MMP-9. J. Investig. Dermatol..

[27] Blakytny R., Jude E. (2006). The molecular biology of chronic wounds and delayed healing in diabetes. Diabet. Med..

[28] Robinson J.M. (2009). Phagocytic leukocytes and reactive oxygen species. Histochem. Cell Biol..

[29] Dissemond J., Goos M., Wagner S.N. (2002). The role of oxidative stress in the pathogenesis and therapy of chronic wounds. Hautarzt.

[30] Mirza R., Koh T.J. (2011). Dysregulation of monocyte/macrophage phenotype in wounds of diabetic mice. Cytokine.

[31] Suntar I., Akkol E., Nahar L., Sarker S. (2012). Wound healing and antioxidant properties: Do they coexist in plants?. Free Rad. Antiox..

[32] Biswas T.K., Mukherjee B. (2003). Plant medicines of indian origin for wound healing activity: A review. Int. J. Low Extrem. Wounds.

[33] Chan M.M. (1995). Inhibition of tumor necrosis factor by curcumin, a phytochemical. Biochem. Pharmacol..

[34] Bierhaus A., Zhang Y., Quehenberger P., Luther T., Haase M., Muller M., Mackman N., Ziegler R., Nawroth P.P. (1997). The dietary pigment curcumin reduces endothelial tissue factor gene expression by inhibiting binding of AP-1 to the DNA and activation of NF-κB. Thromb. Haemost..

[35] Singh S., Aggarwal B.B. (1995). Activation of transcription factor NF-κB is suppressed by curcumin (diferuloylmethane). J. Biol. Chem..

[36] El-Refaie W.M., Elnaggar Y.S., El-Massik M.A., Abdallah O.Y. (2015). Novel curcumin-loaded gel-core hyaluosomes with promising burn-wound healing potential: Development, in vitro appraisal and in vivo studies. Int. J. Pharm..

[37] Heng M.C. (2011). Wound healing in adult skin: Aiming for perfect regeneration. Int. J. Dermatol..

[38] Mohanty C., Das M., Sahoo S.K. (2012). Sustained wound healing activity of curcumin loaded oleic acid based polymeric bandage in a rat model. Mol. Pharm..

[39] Gopinath D., Ahmed M.R., Gomathi K., Chitra K., Sehgal P.K., Jayakumar R. (2004). Dermal wound healing processes with curcumin incorporated collagen films. Biomaterials.

[40] Hegge A.B., Andersen T., Melvik J.E., Bruzell E., Kristensen S., Tonnesen H.H. (2011). Formulation and bacterial phototoxicity of curcumin loaded alginate foams for wound treatment applications: Studies on curcumin and curcuminoides xlii. J. Pharm. Sci..

[41] Friedrich R.B., Kann B., Coradini K., Offerhaus H.L., Beck R.C., Windbergs M. (2015). Skin penetration behavior of lipid-core nanocapsules for simultaneous delivery of resveratrol and curcumin. Eur. J. Pharm. Sci..

[42] Karri V.V., Kuppusamy G., Talluri S.V., Mannemala S.S., Kollipara R., Wadhwani A.D., Mulukutla S., Raju K.R., Malayandi R. (2016). Curcumin loaded chitosan nanoparticles impregnated into collagen-alginate scaffolds for diabetic wound healing. Int. J. Biol. Macromol..

[43] Du L., Feng X., Xiang X., Jin Y. (2016). Wound healing effect of an in situ forming hydrogel loading curcumin-phospholipid complex. Curr. Drug Deliv..

[44] Sidhu G.S., Mani H., Gaddipati J.P., Singh A.K., Seth P., Banaudha K.K., Patnaik G.K., Maheshwari R.K. (1999). Curcumin enhances wound healing in streptozotocin induced diabetic rats and genetically diabetic mice. Wound Repair Regen..

[45] Martin A., Komada M.R., Sane D.C. (2003). Abnormal angiogenesis in diabetes mellitus. Med. Res. Rev..

[46] Kant V., Gopal A., Kumar D., Pathak N.N., Ram M., Jangir B.L., Tandan S.K., Kumar D. (2015). Curcumin-induced angiogenesis hastens wound healing in diabetic rats. J. Surg. Res..

[47] Gadekar R., Saurabh M., Thakur G., Saurabh A. (2012). Study of formulation, characterisation and wound healing potential of transdermal patches of curcumin. Asian J. Pharm. Clin. Res..

[48] Panchatcharam M., Miriyala S., Gayathri V.S., Suguna L. (2006). Curcumin improves wound healing by modulating collagen and decreasing reactive oxygen species. Mol. Cell. Biochem..

[49] Guntas G., Engin B., Ekmekci O.B., Kutlubay Z., Ekmekci H., Songur A., Uzuncakmak T.K., Vehid H.E., Serdaroglu S., Tuzun Y. (2015). Evaluation of advanced oxidation protein products, prooxidant-antioxidant balance, and total antioxidant capacity in untreated vitiligo patients. Ann. Dermatol..

[50] Asawanonda P., Klahan S.O. (2010). Tetrahydrocurcuminoid cream plus targeted narrowband UVB phototherapy for vitiligo: A preliminary randomized controlled study. Photomed. Laser Surg..

[51] Arno A.I., Amini-Nik S., Blit P.H., Al-Shehab M., Belo C., Herer E., Jeschke M.G. (2014). Effect of human wharton’s jelly mesenchymal stem cell paracrine signaling on keloid fibroblasts. Stem Cells Transl. Med..

[52] Hsu Y.C., Chen M.J., Yu Y.M., Ko S.Y., Chang C.C. (2010). Suppression of TGF-β1/smad pathway and extracellular matrix production in primary keloid fibroblasts by curcuminoids: Its potential therapeutic use in the chemoprevention of keloid. Arch. Dermatol. Res..

[53] Song K., Peng S., Sun Z., Li H., Yang R. (2011). Curcumin suppresses TGF-β signaling by inhibition of TGIF degradation in scleroderma fibroblasts. Biochem. Biophys. Res. Commun..

[54] Phan T.T., Sun L., Bay B.H., Chan S.Y., Lee S.T. (2003). Dietary compounds inhibit proliferation and contraction of keloid and hypertrophic scar-derived fibroblasts in vitro: Therapeutic implication for excessive scarring. J. Trauma.

[55] Majtan J. (2014). Honey: An immunomodulator in wound healing. Wound Repair Regen..

[56] Alvarez-Suarez J.M., Gasparrini M., Forbes-Hernandez T.Y., Mazzoni L., Giampieri F. (2014). The composition and biological activity of honey: A focus on manuka honey. Foods.

[57] Jerkovic I., Kus P.M., Tuberoso C.I., Sarolic M. (2014). Phytochemical and physical-chemical analysis of polish willow (*Salix* spp.) honey: Identification of the marker compounds. Food Chem..

[58] Blair S.E., Cokcetin N.N., Harry E.J., Carter D.A. (2009). The unusual antibacterial activity of medical-grade leptospermum honey: Antibacterial spectrum, resistance and transcriptome analysis. Eur. J. Clin. Microbiol. Infect. Dis..

[59] Church D., Elsayed S., Reid O., Winston B., Lindsay R. (2006). Burn wound infections. Clin. Microbiol. Rev..

[60] Molan P.C. (2001). Potential of honey in the treatment of wounds and burns. Am. J. Clin. Dermatol..

[61] Alam F., Islam M.A., Gan S.H., Khalil M.I. (2014). Honey: A potential therapeutic agent for managing diabetic wounds. Evid. Based Complement. Altern. Med..

[62] Hussein S.Z., Mohd Yusoff K., Makpol S., Mohd Yusof Y.A. (2012). Gelam honey inhibits the production of proinflammatory, mediators NO, PGE_2_, TNF-α, and IL-6 in carrageenan-induced acute paw edema in rats. Evid. Based Complement. Altern. Med..

[63] Majtan J., Bohova J., Garcia-Villalba R., Tomas-Barberan F.A., Madakova Z., Majtan T., Majtan V., Klaudiny J. (2013). Fir honeydew honey flavonoids inhibit TNF-β-induced MMP-9 expression in human keratinocytes: A new action of honey in wound healing. Arch. Dermatol. Res..

[64] Hwang H.J., Park H.J., Chung H.J., Min H.Y., Park E.J., Hong J.Y., Lee S.K. (2006). Inhibitory effects of caffeic acid phenethyl ester on cancer cell metastasis mediated by the down-regulation of matrix metalloproteinase expression in human ht1080 fibrosarcoma cells. J. Nutr. Biochem..

[65] Moskwa J., Borawska M.H., Markiewicz-Zukowska R., Puscion-Jakubik A., Naliwajko S.K., Socha K., Soroczynska J. (2014). Polish natural bee honeys are anti-proliferative and anti-metastatic agents in human glioblastoma multiforme U87MG cell line. PLoS ONE.

[66] Henriques A., Jackson S., Cooper R., Burton N. (2006). Free radical production and quenching in honeys with wound healing potential. J. Antimicrob. Chemother..

[67] Gheldof N., Wang X.H., Engeseth N.J. (2002). Identification and quantification of antioxidant components of honeys from various floral sources. J. Agric. Food Chem..

[68] Efem S.E. (1988). Clinical observations on the wound healing properties of honey. Br. J. Surg..

[69] Moghazy A.M., Shams M.E., Adly O.A., Abbas A.H., El-Badawy M.A., Elsakka D.M., Hassan S.A., Abdelmohsen W.S., Ali O.S., Mohamed B.A. (2010). The clinical and cost effectiveness of bee honey dressing in the treatment of diabetic foot ulcers. Diabetes Res. Clin. Pract..

[70] Molan P.C. (2006). The evidence supporting the use of honey as a wound dressing. Int. J. Low Extrem. Wounds.

[71] Pecanac M., Janjic Z., Komarcevic A., Pajic M., Dobanovacki D., Miskovic S.S. (2013). Burns treatment in ancient times. Med. Pregl..

[72] Iftikhar F., Arshad M., Rasheed F., Amraiz D., Anwar P., Gulfraz M. (2010). Effects of acacia honey on wound healing in various rat models. Phytother. Res..

[73] Wijesinghe M., Weatherall M., Perrin K., Beasley R. (2009). Honey in the treatment of burns: A systematic review and meta-analysis of its efficacy. N. Z. Med. J..

[74] Subrahmanyam M. (1991). Topical application of honey in treatment of burns. Br. J. Surg..

[75] Cock I.E. (2015). The medicinal properties and phytochemistry of plants of the genus terminalia (*Combretaceae*). Inflammopharmacology.

[76] Li K., Diao Y., Zhang H., Wang S., Zhang Z., Yu B., Huang S., Yang H. (2011). Tannin extracts from immature fruits of terminalia chebula fructus retz. Promote cutaneous wound healing in rats. BMC Complement. Altern. Med..

[77] Rane M.M., Mengi S.A. (2003). Comparative effect of oral administration and topical application of alcoholic extract of terminalia arjuna bark on incision and excision wounds in rats. Fitoterapia.

[78] Mongalo N.I., McGaw L.J., Segapelo T.V., Finnie J.F., van Staden J. (2016). Ethnobotany, phytochemistry, toxicology and pharmacological properties of *Terminalia sericea* Burch. Ex DC. (*Combretaceae*)—A review. J. Ethnopharmacol..

[79] Zhang H., Zhu S.J., Wang D., Wei Y.J., Hu S.S. (2009). Intramyocardial injection of tannic acid attenuates postinfarction remodeling: A novel approach to stabilize the breaking extracellular matrix. J. Thorac. Cardiovasc. Surg..

[80] Natarajan V., Krithica N., Madhan B., Sehgal P.K. (2013). Preparation and properties of tannic acid cross-linked collagen scaffold and its application in wound healing. J. Biomed. Mater. Res. B Appl. Biomater..

[81] Suguna L., Singh S., Sivakumar P., Sampath P., Chandrakasan G. (2002). Influence of terminalia chebula on dermal wound healing in rats. Phytother. Res..

[82] Singh D., Singh D., Choi S.M., Zo S.M., Painuli R.M., Kwon S.W., Han S.S. (2014). Effect of extracts of terminalia chebula on proliferation of keratinocytes and fibroblasts cells: An alternative approach for wound healing. Evid. Based Complement. Altern. Med..

[83] Reddy D.B., Reddanna P. (2009). Chebulagic acid (CA) attenuates LPS-induced inflammation by suppressing NF-κB and MAPK activation in raw 264.7 macrophages. Biochem. Biophys. Res. Commun..

[84] Soni R., Mehta N.M., Srivastava D.N. (2013). Healing potential of ethyl acetate soluble fraction of ethanolic extract of terminalia chebula on experimental cutaneous wounds in streptozotocin induced diabetic rats. Asian J. Biomed. Pharm. Sci..

[85] Nasiri E., Hosseinimehr S.J., Azadbakht M., Akbari J., Enayati-Fard R., Azizi S. (2015). The effect of terminalia chebula extract vs. Silver sulfadiazine on burn wounds in rats. J. Complement. Integr. Med..

[86] Steenkamp V., Mathivha E., Gouws M.C., van Rensburg C.E. (2004). Studies on antibacterial, antioxidant and fibroblast growth stimulation of wound healing remedies from south africa. J. Ethnopharmacol..

[87] Fyhrquist P., Mwasumbi L., Haeggstrom C.A., Vuorela H., Hiltunen R., Vuorela P. (2002). Ethnobotanical and antimicrobial investigation on some species of terminalia and combretum (*Combretaceae*) growing in tanzania. J. Ethnopharmacol..

[88] Moshi M.J., Mbwambo Z.H. (2005). Some pharmacological properties of extracts of terminalia sericea roots. J. Ethnopharmacol..

[89] Stulberg D.L., Penrod M.A., Blatny R.A. (2002). Common bacterial skin infections. Am. Fam. Phys..

[90] Phan T.T., Hughes M.A., Cherry G.W., Le T.T., Pham H.M. (1996). An aqueous extract of the leaves of *Chromolaena odorata* (formerly eupatorium odoratum) (eupolin) inhibits hydrated collagen lattice contraction by normal human dermal fibroblasts. J. Altern. Complement. Med..

[91] Phan T.T., Hughes M.A., Cherry G.W. (1998). Enhanced proliferation of fibroblasts and endothelial cells treated with an extract of the leaves of *Chromolaena odorata* (eupolin), an herbal remedy for treating wounds. Plast. Reconstr. Surg..

[92] Thang P.T., Patrick S., Teik L.S., Yung C.S. (2001). Anti-oxidant effects of the extracts from the leaves of *Chromolaena odorata* on human dermal fibroblasts and epidermal keratinocytes against hydrogen peroxide and hypoxanthine-xanthine oxidase induced damage. Burns.

[93] Adetutu A., Morgan W.A., Corcoran O. (2011). Antibacterial, antioxidant and fibroblast growth stimulation activity of crude extracts of bridelia ferruginea leaf, a wound-healing plant of Nigeria. J. Ethnopharmacol..

[94] Annan K., Houghton P.J. (2008). Antibacterial, antioxidant and fibroblast growth stimulation of aqueous extracts of *Ficus asperifolia* Miq. And *Gossypium arboreum* L., wound-healing plants of Ghana. J. Ethnopharmacol..

[95] Bahramsoltani R., Farzaei M.H., Rahimi R. (2014). Medicinal plants and their natural components as future drugs for the treatment of burn wounds: An integrative review. Arch. Dermatol. Res..

[96] Duansak D., Somboonwong J., Patumraj S. (2003). Effects of aloe vera on leukocyte adhesion and TNF-α and IL-6 levels in burn wounded rats. Clin. Hemorheol. Microcirc..

[97] Hosseinimehr S.J., Khorasani G., Azadbakht M., Zamani P., Ghasemi M., Ahmadi A. (2010). Effect of aloe cream versus silver sulfadiazine for healing burn wounds in rats. Acta Dermatovenerol. Croat..

[98] Somboonwong J., Thanamittramanee S., Jariyapongskul A., Patumraj S. (2000). Therapeutic effects of *Aloe vera* on cutaneous microcirculation and wound healing in second degree burn model in rats. J. Med. Assoc. Thai..

[99] Rodriguez-Bigas M., Cruz N.I., Suarez A. (1988). Comparative evaluation of *Aloe vera* in the management of burn wounds in guinea pigs. Plast. Reconstr. Surg..

[100] Shahzad M.N., Ahmed N. (2013). Effectiveness of aloe vera gel compared with 1% silver sulphadiazine cream as burn wound dressing in second degree burns. JPMA J. Pak. Med. Assoc..

[101] Visuthikosol V., Chowchuen B., Sukwanarat Y., Sriurairatana S., Boonpucknavig V. (1995). Effect of *Aloe vera* gel to healing of burn wound a clinical and histologic study. J. Med. Assoc. Thai..

[102] Jayakumar R., Prabaharan M., Sudheesh Kumar P.T., Nair S.V., Tamura H. (2011). Biomaterials based on chitin and chitosan in wound dressing applications. Biotechnol. Adv..

[103] Kamoun E.A., Kenawy E.S., Chen X. (2017). A review on polymeric hydrogel membranes for wound dressing applications: PVA-based hydrogel dressings. J. Adv. Res..

[104] Morones J.R., Elechiguerra J.L., Camacho A., Holt K., Kouri J.B., Ramirez J.T., Yacaman M.J. (2005). The bactericidal effect of silver nanoparticles. Nanotechnology.

[105] Boonkaew B., Kempf M., Kimble R., Supaphol P., Cuttle L. (2014). Antimicrobial efficacy of a novel silver hydrogel dressing compared to two common silver burn wound dressings: Acticoat™ and polymem silver^®^. Burns.

[106] Varaprasad K., Vimala K., Ravindra S., Narayana Reddy N., Venkata Subba Reddy G., Mohana Raju K. (2011). Fabrication of silver nanocomposite films impregnated with curcumin for superior antibacterial applications. J. Mater. Sci. Mater. Med..

[107] Merrell J.G., McLaughlin S.W., Tie L., Laurencin C.T., Chen A.F., Nair L.S. (2009). Curcumin-loaded poly(epsilon-caprolactone) nanofibres: Diabetic wound dressing with anti-oxidant and anti-inflammatory properties. Clin. Exp. Pharmacol. Physiol..

[108] Mohd Zohdi R., Abu Bakar Zakaria Z., Yusof N., Mohamed Mustapha N., Abdullah M.N. (2012). Gelam (*Melaleuca* spp.) honey-based hydrogel as burn wound dressing. Evid. Based Complement. Altern. Med..

[109] Nho Y.-C., Park J.-S., Lim Y.-M. (2014). Preparation of hydrogel by radiation for the healing of diabetic ulcer. Radiat. Phys. Chem..

[110] Kawasaki B.T., Hurt E.M., Mistree T., Farrar W.L. (2008). Targeting cancer stem cells with phytochemicals. Mol. Interv..

[111] Dandawate P., Padhye S., Ahmad A., Sarkar F.H. (2013). Novel strategies targeting cancer stem cells through phytochemicals and their analogs. Drug Deliv. Transl. Res..

[112] Kundu J.K., Choi K.Y., Surh Y.J. (2006). β-catenin-mediated signaling: A novel molecular target for chemoprevention with anti-inflammatory substances. Biochim. Biophys. Acta.

[113] Bielefeld K.A., Amini-Nik S., Alman B.A. (2013). Cutaneous wound healing: Recruiting developmental pathways for regeneration. Cell. Mol. Life Sci..

[114] Bielefeld K.A., Amini-Nik S., Whetstone H., Poon R., Youn A., Wang J., Alman B.A. (2011). Fibronectin and β-catenin act in a regulatory loop in dermal fibroblasts to modulate cutaneous healing. J. Biol. Chem..

[115] Amini Nik S., Ebrahim R.P., van Dam K., Cassiman J.J., Tejpar S. (2007). TGF-β modulates β-catenin stability and signaling in mesenchymal proliferations. Exp. Cell. Res..

[116] Amini-Nik S., Cambridge E., Yu W., Guo A., Whetstone H., Nadesan P., Poon R., Hinz B., Alman B.A. (2014). β-catenin-regulated myeloid cell adhesion and migration determine wound healing. J. Clin. Investig..

[117] Amini-Nik S., Glancy D., Boimer C., Whetstone H., Keller C., Alman B.A. (2011). Pax7 expressing cells contribute to dermal wound repair, regulating scar size through a β-catenin mediated process. Stem Cells.

[118] Peters T., Sindrilaru A., Hinz B., Hinrichs R., Menke A., Al-Azzeh E.A., Holzwarth K., Oreshkova T., Wang H., Kess D. (2005). Wound-healing defect of CD18^−/−^ mice due to a decrease in TGF-β1 and myofibroblast differentiation. EMBO J..

[119] Cheon S.S., Wei Q., Gurung A., Youn A., Bright T., Poon R., Whetstone H., Guha A., Alman B.A. (2006). β-catenin regulates wound size and mediates the effect of TGF-β in cutaneous healing. FASEB J..

[120] Cheon S.S., Cheah A.Y., Turley S., Nadesan P., Poon R., Clevers H., Alman B.A. (2002). β-catenin stabilization dysregulates mesenchymal cell proliferation, motility, and invasiveness and causes aggressive fibromatosis and hyperplastic cutaneous wounds. Proc. Natl. Acad. Sci. USA.

[121] Pathania S., Ramakrishnan S.M., Bagler G. (2015). Phytochemica: A platform to explore phytochemicals of medicinal plants. Database.

